# Conformational flexibility and molecular interactions of an archaeal homologue of the Shwachman-Bodian-Diamond syndrome protein

**DOI:** 10.1186/1472-6807-9-32

**Published:** 2009-05-19

**Authors:** C Leong Ng, David G Waterman, Eugene V Koonin, Alison D Walters, James PJ Chong, Michail N Isupov, Andrey A Lebedev, David HJ Bunka, Peter G Stockley, Miguel Ortiz-Lombardía, Alfred A Antson

**Affiliations:** 1 York Structural Biology Laboratory, Chemistry Department, University of York, York, YO10 5YW, UK; 2 Structural Studies Division, MRC Laboratory of Molecular Biology, Hills Road, Cambridge, CB2 0QH, UK; 3 National Center for Biotechnology Information, National Library of Medicine, National Institutes of Health, Bethesda, Maryland, USA; 4 Department of Biology, University of York, York, YO10 5YW, UK; 5 Henry Wellcome Building for Biocatalysis, School of Biosciences, University of Exeter, Stocker Road, Exeter, EX4 4QD, UK; 6 Astbury Centre for Structural Molecular Biology, University of Leeds, Leeds, LS2 9JT, UK; 7 Current address: Architécture et Fonction des Macromolécules Biologiques UMR 9068, Case 932, 163 Avenue de Luminy, 13288 Marseille cedex 9, France

## Abstract

**Background:**

Defects in the human Shwachman-Bodian-Diamond syndrome (SBDS) protein-coding gene lead to the autosomal recessive disorder characterised by bone marrow dysfunction, exocrine pancreatic insufficiency and skeletal abnormalities. This protein is highly conserved in eukaryotes and archaea but is not found in bacteria. Although genomic and biophysical studies have suggested involvement of this protein in RNA metabolism and in ribosome biogenesis, its interacting partners remain largely unknown.

**Results:**

We determined the crystal structure of the SBDS orthologue from *Methanothermobacter thermautotrophicus *(mthSBDS). This structure shows that SBDS proteins are highly flexible, with the N-terminal FYSH domain and the C-terminal ferredoxin-like domain capable of undergoing substantial rotational adjustments with respect to the central domain. Affinity chromatography identified several proteins from the large ribosomal subunit as possible interacting partners of mthSBDS. Moreover, SELEX (Systematic Evolution of Ligands by EXponential enrichment) experiments, combined with electrophoretic mobility shift assays (EMSA) suggest that mthSBDS does not interact with RNA molecules in a sequence specific manner.

**Conclusion:**

It is suggested that functional interactions of SBDS proteins with their partners could be facilitated by rotational adjustments of the N-terminal and the C-terminal domains with respect to the central domain. Examination of the SBDS protein structure and domain movements together with its possible interaction with large ribosomal subunit proteins suggest that these proteins could participate in ribosome function.

## Background

The *Methanothermobacter thermautotrophicus mth685 *gene, which encodes the homologue of the Shwachman-Bodian-Diamond syndrome (SBDS) protein, is located in the predicted exosome superoperon [1]. The SBDS proteins are highly conserved [Pfam:PF01172] in archaea and eukaryota [2]. Mutations of the human *SBDS *gene are associated with the condition known as Shwachman-Diamond syndrome (SDS), an autosomal recessive disorder with clinical features including hematological and skeletal abnormalities and also exocrine pancreatic insufficiency [OMIM:260400]. The most common mutations associated with SDS include the polypeptide chain truncation K62X caused by the introduction of an in-frame stop codon (183–184TA → CT mutation) and a donor splicing site mutation, 258+2T → C, which causes premature truncation of the encoded protein by frameshift (84Cfs3). In addition, several point mutations across the entire sequence of the protein are also associated with SDS [2-6]

Experiments with YLR022c (SDO1), the yeast homologue of the SBDS protein, have shown that its mutations C31W, L71P and I87S affect the protein stability [7]. SDO1 is a non-essential protein [8] present in the cytoplasm, nucleus and nucleolus of the cell [9,10], which is required for G1 cell cycle progression [11]. This protein has been found to interact with 13 different proteins by yeast two-hybrid screening, tandem affinity purification (TAP) and biochemical studies. The set of identified interaction partners include proteins involved in rRNA processing, ribosomal biogenesis, RNA transport, as well as exoribonucleases and two serine/threonine protein kinases [9,12-14]. However, different methods pointed to different interacting partners of SDO1, and none of the hits were retrieved by more than one study. Recently, SDO1 has been shown to contribute to the export of pre-60S ribosomal particles to the cytoplasm by facilitating the release and recycling of the nucleolar shuttling factor Tif6 from the cytoplasmic pre-60S ribosomes [8]. A role in ribosomal biogenesis is further supported by the observed interaction of human SBDS with nucleophosmin (NPM) and Nip7 [15,16].

Other studies have suggested that SBDS proteins might be involved in RNA metabolism. This hypothesis is supported by both its genomic context and by experimental evidence. In particular, the archaeal SBDS orthologues are located in a superoperon that encodes, among others, several proteins of the exosome particle, a conserved multiprotein complex involved in RNA processing and degradation in eukaryotes and archaea [1,17]. Furthermore, the SBDS orthologues in the plants *Arabidopsis thaliana *and *Oryza sativa *contain an extended C-terminal region with putative RNA-binding domains, namely U1-type zinc fingers [7,18].

Hitherto, the only structural information on SBDS proteins is based on two virtually identical X-ray structures of an archaeal orthologue of SBDS from *Archaeoglobus fulgidus *(afSBDS) [7,13]. These studies showed that the SBDS protein contains three domains. The N-terminal FYSH domain (residues 1–86) displays an α/β topology with a novel fold, the middle domain (87–160) has a three-helix bundle fold, and the C-terminal domain (161–234) exhibits a ferredoxin-like fold that is commonly found in proteins with various functions, including numerous RNA binding proteins. Although the protein has an extended tripartite architecture, the extent of its conformational variability was not fully apparent from these structural studies, and its electrostatic or RNA-binding properties have not been characterized either.

Here we address these issues by determining the X-ray structure and probing the RNA and protein-binding capabilities of the SBDS orthologue from the archaeon *M. thermautotrophicus *(mthSBDS). We show that SBDS proteins are highly flexible and can readjust the relative positions of their N-terminal and C-terminal domains in relation to the middle domain. We also present the results of affinity chromatography and SELEX experiments aimed at identifying protein and RNA molecules that interact with the mthSBDS. These data show that the mthSBDS protein can interact with several ribosomal proteins and suggest that it does not interact with a specific RNA sequence. Finally, we discuss the potential functional implications of the flexibility, the shape and the surface charge distribution of mthSBDS.

## Results

### Structure description

**Figure 1:**
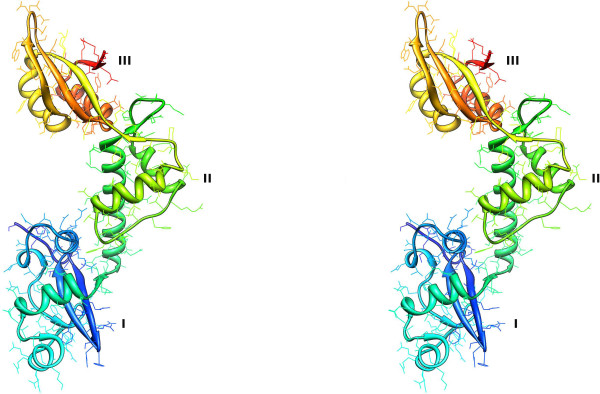
**Ribbon diagram of mthSBDS**. Stereo figure is drawn with colors blending from blue (N-terminus) through to red (C-terminus). Labels indicate domains I, II and III.

**Figure 2:**
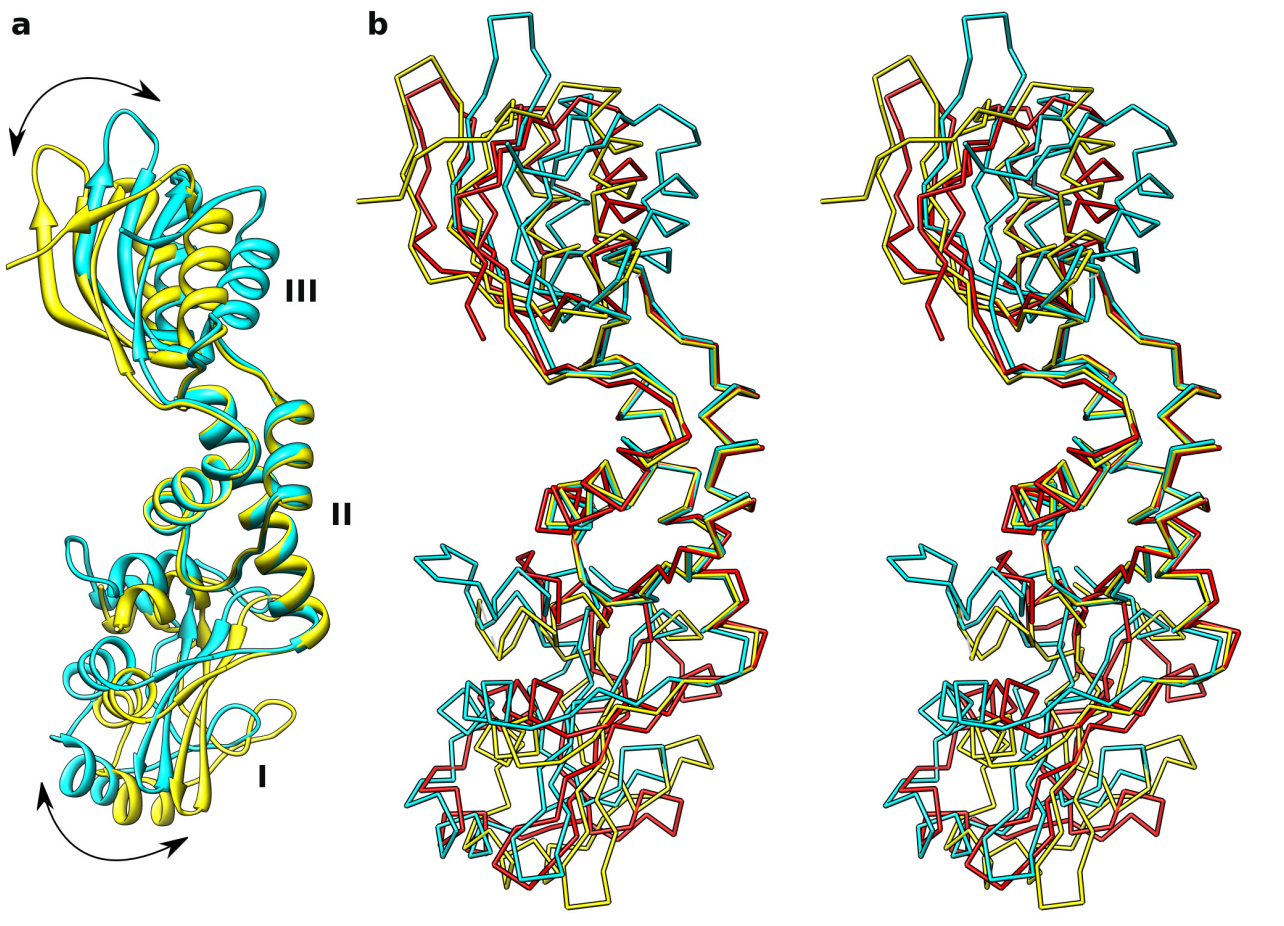
**SBDS protein flexibility**. SBDS molecules are superposed by domain II. The view is as in Figure 1 but rotated by ~90° around the vertical axis. Labels indicate domains I, II and III. a) Ribbon diagrams of two mthSBDS molecules present in the asymmetric unit (A, blue and B, yellow). b) Stereo view of Ca models of the two mthSBDS molecules (A, blue and B, yellow) and afSBDS ([PDB:1P9Q], red).

We obtained crystals of the full-length mthSBDS protein (residues 1–232) belonging to the P222_1 _space group. The X-ray data were collected to a resolution of 1.75 Å and the structure was refined to a final R factor of 19.0% (Rfree of 21.6%) (Table 1). There are two molecules per asymmetric unit (A and B) resulting in a specific volume (V_M_) of 3.2 Å^3^/Da and a solvent content of 61.6%. The model contains residues 1–232, except for residues 33–35 of the second molecule (B) for which there is no clear electron density (Figure 1). The polypeptide chain folds into three domains: domain I (residues 1–88); domain II (residues 89–162) and domain III (residues 163–232). Domain I, also called FYSH domain (for *F*ungal, *Y*hr087w and *Sh*wachman) contains a highly twisted, five-stranded antiparallel β-sheet and four a-helices, all positioned at one side of the sheet. The second domain has a winged helix-turn-helix fold composed of a three-helix, right-handed twisted bundle and a small β-sheet that consists of two or three strands, depending on the stringency of the criteria used for the analysis of the secondary structure. This sheet is shorter than the three or four-stranded β-sheets found in other winged helix-turn-helix domains. Finally, the third domain of mthSBDS protein has the classical ferredoxin-like fold. Although the overall fold of the mthSBDS protein is identical to that of afSBDS (Figure 2), there are significant differences in the relative positioning of the three domains (see below).

**Table 1:** Data collection and refinement statistics

***Data collection***	
Space group	P222_1_

Unit cell, Å	*a *= 68.3, *b *= 72.1, *c *= 146.8; α = β = γ = 90°

Resolution (outer shell), Å^1^	30.0–1.75 (1.75 – 1.78)

Wavelength, Å	1.0081

Completeness, %	98.4 (82.8)

Multiplicity	3.9 (3.0)

Unique reflections	72600 (3002)

<I>/s(I)	20.3 (1.7)

Rmerge^2 ^(%)	6.3 (58.9)

Wilson B factor (Å^2^)	25

***Refinement***	

R-factor^3^/Free R-factor (%)	19.1/21.4

r.m.s.d.^4 ^bonds/angles (Å,°)	0.010 (0.022)/1.173 (1.982)

Average B (Å^2^)	37.1

^1^Values in parenthesis are for the highest resolution shell.

^2^The value of the merging R factor between equivalent measurements of the same reflection, R = Σ_hkl_Σ_j_|I_hkl, j _- <I_hkl_>|/Σ_hkl_Σ_j _I_hkl, j_.

^3^Crystallographic R-factor, R(free) = Σ ||Fo| - |Fc||/Σ |Fo|.

^4^R.m.s. deviation from the standard values are given with target values in parentheses.

In the crystal, molecule B makes extensive interactions with its copy related by crystallographic symmetry. The two molecules have a significant contact area, with ~1400 Å^2 ^per molecule buried in intermolecular contacts. Contacts include 14 direct hydrogen bonds. Four of these bonds are formed between main-chain atoms of the exposed edges of the C-terminal domain β-8 strands, namely between two pairs of residues K164 and R166. Besides a number of van der Waals interactions, up to six plausible ionic interactions contribute to this interface, the most favorable of them between R166 and E205. These extensive intermolecular contacts suggested that the SBDS protein might exist as a dimer. However, sedimentation equilibrium data for the mthSBDS protein showed the presence of only the monomeric form in solution (data not shown). Moreover, no dimer has been observed for any of the available structures of afSBDS, which show different crystal packings. It is also worth noting that only half of the molecules in the crystal of mthSBDS, that is, only molecule B, engage in this interaction. Thus, we conclude that the biological unit of mthSBDS protein is a monomer.

### Structural flexibility of the mthSBDS protein

The presence of two independent molecules in the asymmetric unit provides an opportunity to compare their structures. Surprisingly, there is a substantial difference between their conformations, resulting in an overall r.m.s. difference of 2.5 Å between the backbone atoms (residues 1–232). This is significantly greater than the backbone r.m.s. differences calculated for the three domains separately, which are 0.7 Å (residues 1–88), 0.6 Å (residues 89–162) and 0.8 Å (residues 163–227). Indeed, the high overall r.m.s.d. between the two molecules is due to nearly pure rotations about the hinge regions that can be defined in the boundaries between these domains. Thus, keeping the central domains of the two molecules superimposed, the observed rotations for domains I and III are ~13° and ~29°, respectively (Figure 2a). Interestingly, such substantial conformational adjustments do not lead to major changes in the overall dimensions of the mthSBDS protein.

### Comparison of the mthSBDS and afSBDS proteins

**Figure 3:**
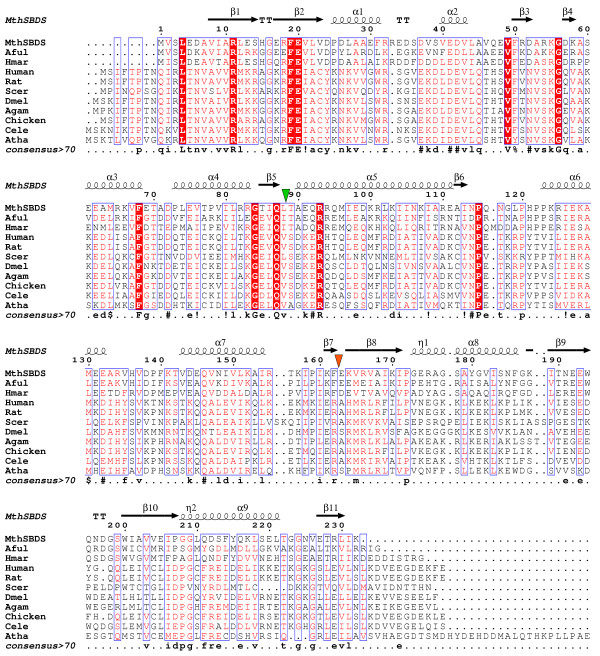
**Sequence alignment of SBDS proteins from different species**. Sequences of SBDS proteins from *M. thermautotrophicus *(MthSBDS), *Archaeoglobus fulgidus *(Aful), *Halobacterium marismortui *(Hmar), *Saccharomyces cerevisiae *(Scer), *Human*, *Rat*, *Chicken*, *Anopheles gambiae *(Agam), *Drosophila melanogaster *(Dmel), *Caenorhabditis elegans *(Cele) and *Arabidopsis thaliana *(Atha) were aligned. Green and orange triangles designate boundaries between domains 1 and 2 and domains 2 and 3, respectively. The secondary structure of mthSBDS (molecule A) is shown on top of the aligned sequences. Positions for which the percentage of 'equivalent' residues, considering their physico-chemical properties, is higher than 70% are colored in red on a white background and framed in blue boxes. If residues are strictly conserved, they are colored in white characters on a red background.

The mthSBDS protein shares 50% and 25% sequence identities with its *A. fulgidus *and human orthologues, respectively. A structure-based sequence alignment of mthSBDS and afSBDS protein domains shows that domains I and II are more conserved than domain III (sequence identities of 51%, 55% and 41%, respectively). A similar pattern of conservation is observed in the alignment of all members of the SBDS family, as defined by [Pfam:PF01172], with overall sequence similarities of 33%, 30% and 14% (BLOSUM62 substitution matrix), respectively, for domains I, II and III (see Figure 3).

Comparison of the two independent copies of mthSBDS present in the asymmetric unit with the available structures of afSBDS demonstrates differences in the relative orientation of the three domains (Figure 2b). The main-chain r.m.s. difference between the full-length proteins mthSBDS (chain A) and afSBDS ([PDB: 1P9Q, PDB:1T95]) is 3.3 Å. Again, superposition of individual domains results in lower main-chain r.m.s. differences: 1.3 Å (domain I), 1.0 Å (domain II) and 1.8 Å (domain III). These figures correspond to comparison of mthSBDS with [PDB: 1P9Q], but similar differences are found when comparison is made with the [PDB: 1T95] structure (main-chain r.m.s.d between the [PDB:1P9Q] and [PDB:1T95] structures is 0.4 Å). Thus, hereafter our discussion will be based on comparisons between mthSBDS and the [PDB:1P9Q] structure.

We analyzed the changes in the relative orientations of domains I and III with respect to domain II between mthSBDS (molecule A) and afSBDS. When the central domains of the two molecules are superimposed, the observed rotations for domains I and III are ~16° and ~23°, respectively. The directions of the rotation axes are not coincident with those found between the two copies of mthSBDS in the asymmetric unit, indicating that apart from rotational rearrangement, the individual domains may undergo limited translational adjustment. Nevertheless, the hinge regions between the domains are the same. This observation is thus consistent with the domain rotations found for the two molecules of mthSBDS present in the asymmetric unit.

### mthSBDS interacts with ribosomal proteins

**Figure 4:**
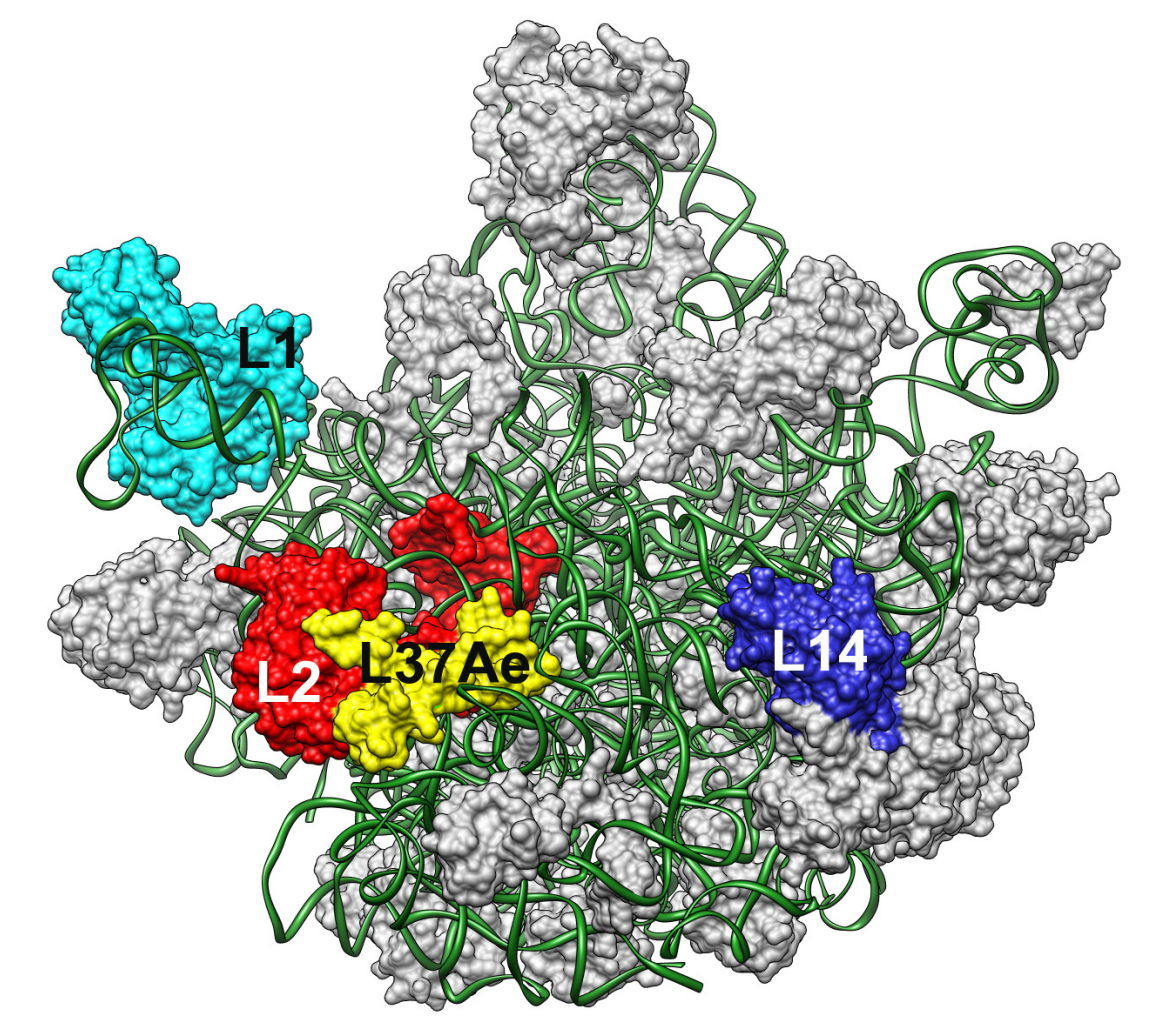
**Location of L1, L2, L14 and L37AE proteins within the *Haloarcula marismortui *50S ribosomal subunit **[PDB:1JJ2, **PDB:**1MZP]. rRNA is shown as a ribbon (green) and proteins are represented by their molecular surfaces. The three proteins found to interact with mthSBDS and the ribosomal L37AE protein encoded within the exosome superoperon are labeled and colored.

To gain insight into the function of mthSBDS, we carried out a series of affinity chromatography experiments. Cell lysates from *M. thermautotrophicus *were digested with nucleases and loaded onto mthSBDS-coupled NHS-activated affinity columns. The bound proteins were then eluted at acidic pH. The eluted proteins were separated in SDS-polyacrylamide gels, silver stained and the specific bands excised and examined by mass spectrometry (Additional file 1). Analysis of the mass spectrometric data, carried out with the MASCOT software, gave five significant hits to three proteins, which were identified as the large ribosomal subunit components L1, L2 and L14 (Figure 4, Table 2).

**Table 2:** Summary of MASCOT analysis of affinity chromatography eluted material

*M. thermautotrophicus*	Protein score	Protein	Total ion	Total
Protein/Uniprot ID		C.I. %^1^	score	C.I. %^1^
Ribosomal protein L2/[UniProt:O26113]	251	100	148	100
Ribosomal protein L14/[UniProt:O26121]	212	100	130	100
Ribosomal Protein L1/[UniProt:O27716]	283	100	155	100

The results of the search of the NCBI non-redundant database using combined MS-MS/MS data obtained by MALDI-TOF/TOF mass spectrometry. Proteins were excised from gels after SDS-PAGE of fractions eluted from a column carrying immobilised mthSBDS.

^1^Confidence interval

### mthSBDS does not bind to specific RNA sequences

In order to identify any sequence-specific interactions with RNA ligands, an *in vitro *selection experiment was performed with a degenerate library of approximately 10^15 ^sequences. After 10 rounds of robotic selection carried out as previously described [19], the aptamer pool was subjected to a mobility shift assay. This resulted in the appearance of a shifted RNA species in a protein concentration-dependent manner, at mthSBDS concentrations higher than ~5 μM (Additional file 2a). However, a similar shift at ~5 μM mthSBDS was also seen when the experiment was repeated using the naïve starting pool of RNA. This suggests that the selections did not lead to any improvement in RNA-binding affinity, consistent with the conclusion the bound RNA pools contained no strong conserved sequence motifs (Additional file 2b). Likewise, comparison of the predicted secondary structures of the enriched RNA sequences failed to detect any significant folding similarity. These data are consistent with mthSBDS not binding RNA in a sequence-specific way. Notwithstanding, we tested binding of mthSBDS protein to one individual aptamer present in the final pool of selected RNA segments. The individual aptamer selected contained the closest sequence to a consensus (A-G-C-C-----A-T) (Additional file 2b). We used a 30-base poly(A) RNA as a control. Neither electrophoretic mobility shift assays nor Surface Plasmon Resonance experiments detected significant interactions with these RNAs (data not shown). Taken together, these experiments indicate, within the limits of the SELEX method, that mthSBDS protein does not bind to a specific sequence of RNA.

## Discussion

### Similarities and differences in the surface properties of archaeal and eukaryotic FYSH domains

Amino acid substitutions of several conserved residues in the N-terminal FYSH domain of human SBDS have been linked with the SDS condition [2,18,20] suggesting that this domain plays an important physiological role. Indeed, the FYSH domain has the most conserved sequence among archaeal and eukaryotic SBDS proteins (Figure 3) resulting in similar features being exposed on its surface. However, the archaeal FYSH domains are somewhat shorter and have fewer conserved residues than their eukaryotic counterparts (12 compared to 25).

**Figure 5:**
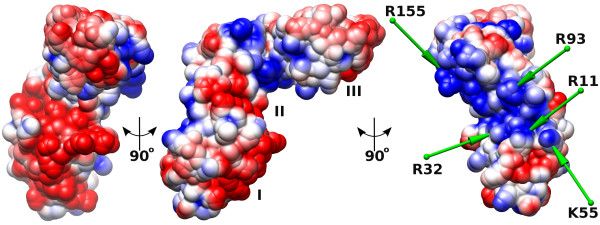
**Surface electrostatic potential of mthSBDS**. Three different views, each 90° apart along the vertical axis of the figure, are shown with colors ranging from red (-0.5 V) to blue (0.5 V). Labels indicate domains I, II and III. The electrostatic potential was calculated using the PARSE forcefield at an implicit ionic strength of 150 mM NaCl and mapped on the solvent accessible surface of mthSBDS. The FYSH domain is at the bottom of the figure. On the right panel, the positions of selected positively charged residues are indicated with green arrows (see text for details).

One side of the FYSH domain contains multiple Asp and Glu residues, generating a large surface area with an overall negative charge (Figure 5). Most of the amino acids contributing to this surface area are highly conserved among archaeal and eukaryotic SBDS proteins (Figure 3), in particular, E20, D37, E41, D42 and D72. Indeed, this domain has an overall acidic pI in archaeal proteins (~4.5 for mthSBDS). Interestingly, despite the conservation of these negatively charged residues, FYSH domains of eukaryotic SBDS proteins usually display an overall positive charge (pI ~9). This is due, on the one hand, to the presence of a series of basic amino acids, up to six of them among the first 25 residues of the eukaryotic FYSH domain, which are not conserved in the archaeal sequences. On the other hand, archaeal SBDS proteins show a higher content in acidic amino acids (for example, 22 in the mthSDBS vs. 13 in the human SBDS). If, as expected, the overall architecture of the domain is preserved in eukaryotes, their characteristic basic residues would be clustered at the surface opposite to the negatively charged area defined by the ubiquitously conserved acidic residues. This would result in a remarkable polarity of the domain that is much less pronounced in archaea (Figure 5). For example, mthSBDS includes only three positively charged residues in this region, namely R11, R32 and K55. The polarity in the surface charge distribution suggests that the two sides of the FYSH domain could be functionally important, possibly involving protein-protein interactions at the negatively charged surface and interactions with the nucleic acid phosphate backbone via the positively charged area. The functional importance of one of the positively charged residues, the universally conserved R11, is compatible with the detection of a R → Q substitution at this position (R19Q in the human SBDS) in SDS patients [2].

The different charge distribution observed in the archaeal FYSH domains hints at the possibility that eukaryotic and archaeal SBDS proteins might have evolved to play somewhat different cellular roles. It is therefore not surprising that yeast complementation assays have revealed that FYSH domains are interspecies exchangeable among eukaryotes, but not between archaea and eukarya [2]. Alternatively, it is also possible that SBDS proteins and their partners have co-evolved to display different surface properties despite functional conservation.

### Domains II and III have ubiquitous folds compatible with different functions

According to Dali [21] and SSM [22], the three-helix bundle of domain II is similar to the fold of UBA-like domains and to C-terminal domain domain III of the Holliday-junction binding protein RuvA [SCOP:46928]. However, the similarity is restricted to the three a-helices and does not extend to the small β-sheet that is present in domain II of mthSBDS. Although the function of UBA (Ubiquitin Associated) domains is not fully understood, they seem to be involved in protein-protein recognition via a hydrophobic patch on their surface [23]. In RuvA, domain III does not have any direct interaction with DNA nor does it contribute to DNA recognition. Instead, it might play a role in the ATP-dependent branch migration through direct contacts with RuvB. None of the proteins with high similarity scores to domain II of mthSBDS seems to bind DNA via the three-helical bundle. However, the surface properties of domain II of SBDS suggest its possible involvement in direct interactions with nucleic acids. Unlike domain III of RuvA that has an overall negative charge (pI ~5.2) or the hydrophobic UBA domains, the central domain of archaeal and eukaryotic SBDS proteins has a positive overall charge (pI ~10). The basic pI ensues from several arginine residues, namely R93 and R155 in mthSBDS, which are conserved in both archaea and eukaryotes. These residues could be involved in interactions with nucleic acids (Figure 5). Another arginine, the non-conserved R101, appears to preserve the correct positioning of helices a5 and a6 in domain II via ionic interactions with E127 and E98.

Domain III of mthSBDS has the ferredoxin-like fold that is found in many RNA-binding proteins and is most similar to the domain V of the EF-G and EF-2 proteins according to an SSM search [22]. RNA-binding proteins with such a fold usually bind the cognate nucleic acid molecules at the surface of their four-stranded β-sheet [24]. However, the sequence identity between domain III of mthSBDS and the most structurally similar RNA-binding domains is relatively low (~20%) and, more importantly, the functionally important RNA-binding residues are not conserved in the mthSBDS (data not shown). The sequence alignment of SBDS proteins from different organisms (Figure 3) reveals that residues which have their side chains exposed at the surface of domain III are the least conserved, even among the SBDS proteins from closely related organisms. The variability of the surface residues suggests that domain III is not likely to be involved in specific interactions. Nevertheless, this domain appears to possess an important function. Indeed, a yeast complementation analysis showed that, although deletion of domain III did not abrogate growth [2], it led to quantitative growth defects [7]. The functional importance of this domain is further supported by the identification of SDS patients with the point mutation R175W [3]. Although the exact effect of this substitution is unknown, inspection of the structure shows that it could cause a major change in the fold of the domain III and hence alter its shape. Another substitution associated with SDS, R218stop, would result in a shorter polypeptide [6,20], affecting the overall size of domain III. Taken together, the data indicate that while the nature of the residues at the surface of domain III is not critical for the proper function of the protein, this domain plays an important structural role, which depends on its overall shape and size. In this respect, the exposed edges of domain III β-sheet, which are lined up by main chain atoms of β-strands β9 and β11, may play a role in interaction. On the one hand, strand β9 carries the EEW motif, which appears to be the most conserved sequence feature among domains III of the SBDS molecules from different species (Figure 3). On the other hand, the potential for strand β11 to engage in interactions is demonstrated by its contacts with the equivalent β-strand of a symmetric mthSBDS molecule in the crystal. Though this interaction would not be sufficient for the formation of an SBDS dimer in solution, mthSBDS might bind to a possible partner via this kind of β-β interaction.

### SBDS proteins are intrinsically flexible

Our finding that the two molecules of mthSBDS observed independently in the crystal adopt somewhat different conformations, which in turn differ from that adopted by afSBDS, prompted us to further investigate possible conformational changes by normal mode analysis calculations using the ElNémo server [25]. The low energy normal modes generally describe the most collective movements of a protein. Moreover, when the structure of a protein is known in two different conformations, comparison of the observed conformational differences with the results of normal mode analysis may identify which particular modes contribute most significantly to conformational changes.

**Figure 6:**
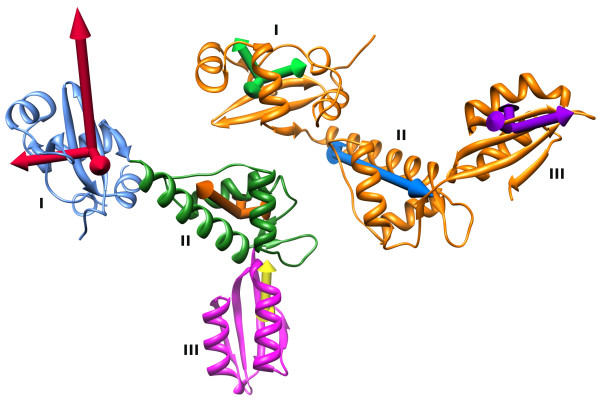
**Analysis of mthSBDS TLS groups**. The asymmetric unit of the mthSBDS crystal is represented in ribbons with molecule A in orange and molecule B colored by domains: domain I in blue, domain II in green and domain III in magenta. The principal axes of libration of each TLS group are represented as arrows; their lengths are proportional to the mean libration along them.

We found that three non-trivial, low frequency modes described more than 70% of the observed conformational change between molecules A and B in the crystal structure of mthSBDS (modes 9, 10 and 11). The largest contribution results from mode 11, which on its own contributes to 28% of the motions. Moreover, three among the first 10 non-trivial modes are sufficient to describe 64% of the conformational change between mthSBDS molecule A and afSBDS. In this case, a single mode (mode 8) contributes more than 40% to the inferred motion. Indeed, perturbation of the structure of mthSBDS molecule A along mode 8 causes the FYSH and C-terminal domains to rotate with respect to the central domain (Additional file 3). Such rotational adjustments are in agreement with the observed conformational differences between the A and B molecules of mthSBDS and the molecule of afSBDS (Figure 2). Furthermore, such movements also agree with the directions of the principal axes of libration (Figure 6) calculated by TLSANL [26] following the refinement of mthSBDS structure using the translation-libration-screw (TLS) approximation, with each of the three domains of the two molecules treated as independent TLS groups.

Taken together, these observations indicate that SBDS proteins are intrinsically flexible and that displacement along a few normal modes could be sufficient to bring about large, possibly, functionally relevant structural transitions.

The intrinsic flexibility of the mthSBDS could help explain why the apparently strong dimer present in the crystal (see Results) is not found in solution. Under these circumstances, the formation of the dimer would be entropically disfavored.

### mthSBDS, like other SBDS proteins, might interact with ribosomes

In the absence of a detailed understanding of the functions of SBDS proteins, we can only speculate about the potential roles of the structural rearrangements that we have demonstrated that these proteins might undergo. In this regard, we can gain some insight by considering the possible interaction partners of SBDS proteins. The three proteins interacting with mthSBDS protein that were identified in the present study by affinity chromatography are all from the large ribosomal subunit. These data give further support to an earlier study where the yeast SBDS protein (SDO1) co-purified with components of the 60S ribosomal particle [13]. The data are also consistent with the recent discovery that SDO1 is involved in the maturation of the pre-60S large ribosomal subunit [8].

Since cell lysates were digested with RNAse prior to their loading onto the affinity columns, ribosomal proteins would be exposed to mthSBDS either as separate RNA-free proteins or as smaller protein-protein complexes. Therefore, the binding of these proteins to mthSBDS should be informative about the putative interacting surfaces between the mthSBDS and the ribosome. Within the large ribosomal subunit, the three interacting components of mth685 protein, namely the L1, L2 and L14, do not interact directly with each other (Figure 4). Each of these proteins has a significant amount of solvent exposed surface, making interactions with external factors a physical possibility. L1 interacts solely with the 23S rRNA and is separated from the main body of the particle. L2 is the largest protein component of the large ribosomal subunit and is found close to L1. Interestingly, L2 closely associates with another ribosomal protein L37AE, which, like mthSBDS protein, is encoded within the exosome superoperon. Structural observations described above indicate that interactions between mthSBDS and these proteins, or their neighbors in the ribosome, could be assisted by the extended structure and flexibility of the SBDS proteins that allows adjustments in the relative position and orientation of individual domains.

In this context, it may be interesting to note that the size and shape of the mthSBDS protein are remarkably similar to those of tRNA. Indeed, one arm of the L-shaped mthSBDS protein is ~58 Å long comprising domain I and part of domain II. The length of the second arm, formed by the rest of domain II and domain III, is ~56 Å. Furthermore, a number of negatively charged patches, especially in domains I and II of mthSBDS, match the negatively charged phosphates on the surface of a tRNA molecule (Figure 5). However, several positively charged residues on the surface of mthSBDS have no match on the surface of tRNA. Further experiments should address the question of whether these similarities play a role or not on mthSBDS function.

## Conclusion

We have determined the X-ray structure of the SBDS protein from *Methanothermobacter thermautotropicus *(mthSBDS). Structural observations reveal the intrinsic flexibility of SBDS proteins, a feature that we hypothesize to have direct implications for their functions. In keeping with previous results published for other SBDS homologs, we found, by means of affinity chromatography capture, that mthSBDS can interact with ribosomal proteins whereas RNA-SELEX experiments suggest it does not interact specifically with RNA. Involvement of the mthSBDS protein in the function and/or maturation of the translation machinery is possible, as previously suggested for SBDS proteins from other organisms [8]. We propose that the flexibility of SBDS molecules might allow them to adjust their functionally important interactions with the similarly flexible ribosome to fulfill their function.

## Methods

### Cloning

The *Mth685 *gene from *Methanothermobacter thermautotrophicus *was amplified by PCR from genomic DNA with primers 5'-GAG GAG TAA CAT ATG GTC AGC CTT GAA GAT G-3' (forward) and 5'-AGG AGA GGA AGC TTA TTT TAT TAG CCT GGT TTC AAC-3 (reverse). The PCR product was cloned into the pET28a expression vector (Novagen, Madison, WI, USA), using NdeI and HindIII restriction sites to produce plasmid pYCL02. The clone was sequenced and found to be identical to the published sequence except for nucleotide 534, which was changed from G to T, resulting in the R175M substitution. Expression from pYCL02 yielded a recombinant mthSBDS protein which, in addition to 232 residues encoded by the cloned DNA, contained a 19 amino acid-long N-terminal extension with a (His)_6 _tag.

### Protein expression, purification and crystallisation

An overnight culture of pYCL02-transformed *Escherichia coli *Rosetta (DE3) cells was used to inoculate fresh Lysogeny Broth (LB) medium. The cells were grown at 37°C to ~0.6 OD_600_, then moved to 16°C and induced for overnight expression with 1 mM isopropyl-β-D-thiogalactoside. The bacterial pellet was resuspended and the cells were sonicated in a solution containing 50 mM Tris (pH 7.5), 0.5 M NaCl and protease inhibitors. The lysate was cleared by centrifugation and syringe filtration through 0.2 μm filters (Sartorius). The supernatant was applied to Ni^2+ ^loaded HisTrap columns (Amersham Biosciences) for affinity chromatography. Fractions containing the mthSBDS protein were pooled and further purified by gel filtration in a Superdex 200 prep grade column (Amersham Biosciences) equilibrated with 25 mM Tris (pH 7.5) and 0.1 M NaCl. The protein was subsequently concentrated to ~22.5 mg/ml.

Crystals of mthSBDS protein were obtained by hanging-drop vapour diffusion at 19°C, using (1 + 1) μl (protein + reservoir) against a reservoir including 0.9 M lithium sulphate, 0.5 M ammonium sulphate and 0.1 M sodium citrate (pH 5.6). Before flash-cooling in liquid nitrogen, crystals were transferred into a cryo-protectant solution containing 28% glycerol in addition to crystallisation reagents.

### Crystal structure determination and refinement

Diffraction data were collected at BM14, ESRF, Grenoble and processed to a resolution of 1.75Å using DENZO/SCALEPACK [27]. Merging statistics are summarized in Table 1. The structure, including two molecules of mthSBDS protein in the asymmetric unit, was determined by MOLREP [28] from the CCP4 suite [29] using the structure of the afSBDS protein (Protein Data Bank code 1P9Q) as search model. Although mthSBDS protein is closely homologous to the afSBDS protein (49% sequence identity), it was not possible to find a molecular replacement solution when the whole protein was used as the search model. Therefore, we dissected the reference model [PDB:1P9Q] into three domains (Domain I: residues 1–89; Domain II: residues 90–162 and Domain III: residues 163–231) and performed molecular replacement searches using these separate domains. The best solution was obtained for domain I of one of the two molecules (hereafter designated as molecule A) in the asymmetric unit. The solution for this domain was improved by rigid body refinement and was fixed during further molecular replacement searches. The second best solution was obtained for domain II of molecule B. At this stage rigid body refinement was applied to both domain solutions and was followed by 10 cycles of all-atoms restrained refinement using REFMAC [30]. At the next step, both refined domains were fixed, and the next best molecular replacement solution found was for domain II of molecule A. This procedure of refinement/molecular replacement search was iterated until solutions for all three domains of both molecules were found. The final molecular replacement model was further rebuilt and improved by use of ARP/WARP followed by cycles of manual model rebuilding with COOT [31] and restrained refinement, including TLS option in REFMAC.

For the purpose of projecting the normal modes onto the observed displacements between molecules A and B in the crystal of mthSBDS and between mthSBDS molecule A (mthSBDS-A) and the structure of afSBDS, the domains of mthSBDS-A were superimposed on the respective domains of the other molecules using the least-squares procedure in LSQKAB [32]. The domains were then joined together and the resulting chimeric structure superimposed back onto mthSBDS-A. This procedure ensures the presence of the same set of atoms in the two conformations analyzed by ElNémo.

The atomic coordinates and structure factors have been deposited in the Protein Data Bank [PDB:2WBM].

### Affinity chromatography

*M. thermautotrophicus *cells were grown as previously described [33] and resuspended in 1.5 ml/g (wet cells) of resuspension buffer (20 mM Tris pH 8, 1 mM EDTA, 10% glycerol, 1 mM DTT, 0.5 M KOAc). Phenyl-methane-sulphonyl fluoride (PMSF) was added to a final concentration of 0.1 mM and cells were lysed using a French's press at 10,000 psi. Nucleases RNase A (Roche) and DNase I (Sigma) were added to a final concentration of 10 μg/ml and incubated at room temperature for 30 min. The cell lysates were centrifuged at 15000 *g *for 25 minutes at 4°C and supernatants snap-frozen in 1 ml aliquots and stored at -80°C. 3 mg of mthSBDS recombinant protein was coupled to a 1 ml NHS-activated column (GE Healthcare). A control column was coupled with 3 mg of BSA. Coupling efficiency was assessed using PD-10 desalting columns (GE Healthcare). After the cell extracts were loaded to the column containing covalently cross-linked protein, bound proteins were eluted with 3 ml of 100 mM glycine-HCl at pH 2. Fractions of 200 μl were collected into 20 μl of 2 M Tris pH 8 in order to neutralize the pH. They were then concentrated using Deoxycholate/Tri-chloro-acetic acid (DOC-TCA) precipitation and separated by SDS-PAGE before being visualized using silver staining. Protein bands of interest (those that eluted from the test column but did not elute from the control column) were excised, digested with trypsin [34] and analyzed by mass spectrometry. Mass spectrometric analysis was carried out using a 4700 Proteomics Analyzer (Applied Biosystems). The MASCOT software (Matrix Science Ltd.) was used to search the NCBI non-redundant database with combined MS and MS/MS data.

### Identification of possible specific RNA partners by Systematic Evolution of Ligands by EXponential enrichment (SELEX)

#### a) in vitro selection of RNA aptamers and PCR

A degenerate library of ~10^15 ^RNA sequences was used in 10 rounds of *in vitro *selection against recombinant mthSBDS in 25 mM Tris pH 7.5 and 0.5 M NaCl. The 99 nucleotide library has the following sequence:5'-GA**TAATACGACTCACTATAGGGAA**TGGATCCACATCTACGAATTC-N_30_-TTCACTGCAGACTTGACGAAGCTT-3'

It contains two constant regions flanking the N_30 _variable locus. The constant region at the 5' end includes a T7 RNA polymerase promoter sequence (**bold**) for *in vitro *transcription purposes. The 5' and 3' constant region sequences were used as specific primers for the library. The 10 round selection of RNA aptamers pools were carried out using automated selection method as described previously [19].

#### b) RNA synthesis and labeling

RNA samples were prepared in a two-step transcription reaction. 2 μl 10× transcription buffer, 1 μl RNAsecure™ (Ambion inc.), 1 μl 1 M DTT, 2.6 μl RNase free water and 2 μl template DNA were mixed and heated at 60°C for 20 minutes to activate the RNAsecure™. Reactions were then continued by adding 2.4 μl 100 μM UTP, 1 μl 10 mM of CTP, GTP and ATP, 20 units of RNaseOUT™ (Invitrogen), 100 units of T7 RNA polymerase (Roche) and 50 μCi a-^32^P UTP (3000 Ci mmol^-1^, 10 μCi μl^-1^). The reaction samples were incubated at 37°C for 90 minutes. The labeled RNA samples were purified by treatment with DNase 1 (Invitrogen), followed by acidified phenol/chloroform extraction and ethanol precipitation. The pellets were dried and resuspended in 50 μl of RNase-free water.

#### c) Gel mobility shift assays

The gel mobility shift assay was used to quantitatively measure the affinity of mthSBDS-RNA binding. In this assay, protein concentration ranged from 10^-4 ^M to 10^-12 ^M and the ^32^P-radio-labelled RNA was added in 1% v/v quantities of total transcribed RNA sample. The shifted RNA species were excised, eluted and reverse transcribed to generate cDNA sequences of the selected RNAs. The DNAs were cloned into pGEM-T Easy vector (Promega) and sequenced (York Bioscience). Consensus sequence was generated using CLUSTALW (EBI) and RNA secondary structure was predicted using MFOLD [35].

#### d) Surface Plasmon Resonance

Surface Plasmon Resonance experiments were performed using a BIAcore T100 system. The mthSBDS protein, at a concentration of 0.25 μM was used to label the Ni^2+ ^loaded NTA sensor chips before the analysed RNA (1 μM or 50 μM, both with and without 5 mM MgCl_2_) was applied. The buffer used in these experiments contained 10 mM HEPES pH 7.5, 150 mM NaCl, 5 μM EDTA and 0.05% P20.

### Sequence and structure analysis

Sequences were aligned with MUSCLE [36] and the alignment was represented using ESPRIPT [37]. The program CHIMERA [38] was used for the visual inspection of structure properties and the generation of molecular representation figures. Electrostatic potential analyses were carried out with APBS [39].

## Authors' contributions

CLN cloned the mth685 gene, produced and crystallized the mthSBDS protein, refined the structure of mthSBDS, carried out the SELEX experiments, participated in the affinity chromatography assays and drafted the manuscript. DGW and EVK performed the bioinformatics analysis that led to the selection of the mth685 gene, they also contributed to the design of the experiments and to the discussion of their results. JPJC designed the affinity chromatography assays and ADW made the major contribution to these assays. MNI and AAL solved the structure of mthSBDS. DHJB and PGS designed the SELEX experiments and DHJB helped carrying them out. MOL carried out the analysis of the flexibility of the mthSBDS protein. MOL and AAA conceived the study, participated in the design of the experiments, helped with the refinement of the structure and coordinated the writing of the manuscript. All authors read and approved the final manuscript.

## Supplementary Material

Additional File 1 **Identification by affinity chromatography of possible mthSBDS protein partners**. a) Silver-stained SDS-polyacrylamide gel of fractions eluted from a negative control column coupled with BSA. b) Silver-stained SDS-polyacrylamide gel analysis of fractions eluted from a column coupled with 3 mg of mthSBDS. Ribosomal proteins L2 and L14 were identified by mass spectrometry from band 1, while L1 was detected in band 2. Band 3 did not lead to the unambiguous identification of a particular protein sequence. Click here for file

Additional File 2 **SELEX results**. a) Mobility shift assay of mthSBDS protein with radio-labelled RNA. Protein concentration ranges: 1: 10^-4 ^M, 2: 10^-5 ^M, 3: 10^-6 ^M, 4: 10^-7 ^M, 5: 10^-8 ^M, 6: 10^-9 ^M, 7: 10^-10 ^M, 8: 10^-11 ^M, 9: 10^-12 ^M, 10: no protein. b) Sequence alignment of 20 DNA sequences of RNA species recovered from mobility shift assay. The most conserved residues in the consensus sequence are indicated by red dots. The boxed RNA sequence was tested for binding to mthSBDS by Surface Plasmon Resonance. Click here for file

Additional File 3 **Movie showing the conformational flexibility of mthSBDS**. In this movie, intermediate models (yellow) were generated by the ElNémo server (mode 8, amplitudes ranging from 10 to 150 in arbitrary units) using molecule A (blue) of mthSBDS. The structure of afSBDS [PDB:1P9Q] (green) is superimposed with that of mthSBDS. Click here for file
