# Supplementary figures and images for: Conformational flexibility and molecular interactions of an archaeal homologue of the Shwachman-Bodian-Diamond syndrome protein
Source: BMC Struct Biol. 2009 May 19;9:32. doi: 10.1186/1472-6807-9-32 (PMC2695463; doi:10.1186/1472-6807-9-32)

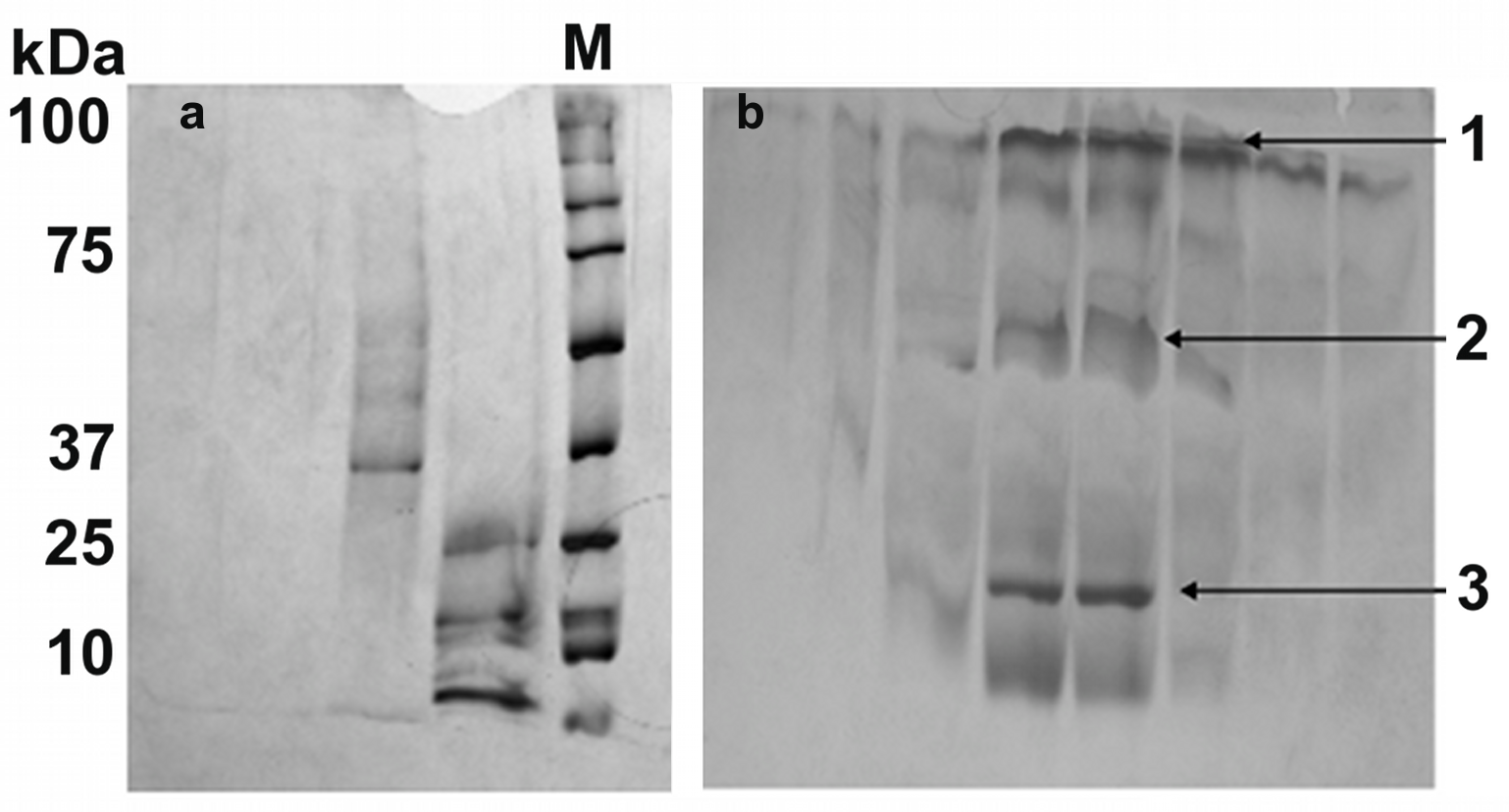

Supplement: Additional File 1 — Identification by affinity chromatography of possible mthSBDS protein partners. a) Silver-stained SDS-polyacrylamide gel of fractions eluted from a negative control column coupled with BSA. b) Silver-stained SDS-polyacrylamide gel analysis of fractions eluted from a column coupled with 3 mg of mthSBDS. Ribosomal proteins L2 and L14 were identified by mass spectrometry from band 1, while L1 was detected in band 2. Band 3 did not lead to the unambiguous identification of a particular protein sequence. [file 1472-6807-9-32-S1.png]

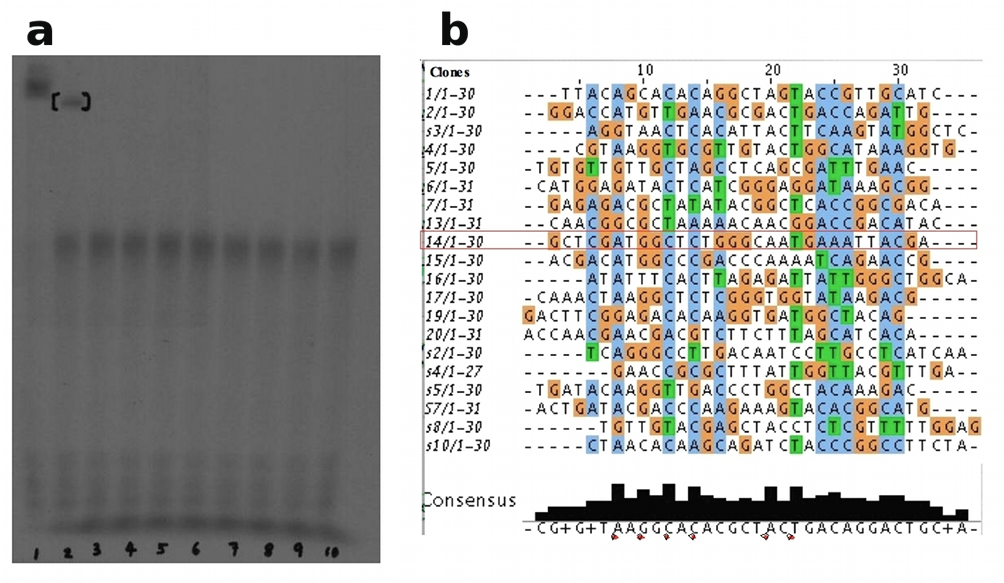

Supplement: Additional File 2 — SELEX results. a) Mobility shift assay of mthSBDS protein with radio-labelled RNA. Protein concentration ranges: 1: 10-4 M, 2: 10-5 M, 3: 10-6 M, 4: 10-7 M, 5: 10-8 M, 6: 10-9 M, 7: 10-10 M, 8: 10-11 M, 9: 10-12 M, 10: no protein. b) Sequence alignment of 20 DNA sequences of RNA species recovered from mobility shift assay. The most conserved residues in the consensus sequence are indicated by red dots. The boxed RNA sequence was tested for binding to mthSBDS by Surface Plasmon Resonance. [file 1472-6807-9-32-S2.png]
